# Simultaneous Biofortification of Rice With Zinc, Iodine, Iron and Selenium Through Foliar Treatment of a Micronutrient Cocktail in Five Countries

**DOI:** 10.3389/fpls.2020.589835

**Published:** 2020-11-13

**Authors:** Chanakan Prom-u-thai, Abdul Rashid, Hari Ram, Chunqin Zou, Luiz Roberto Guimaraes Guilherme, Ana Paula Branco Corguinha, Shiwei Guo, Charanjeet Kaur, Asif Naeem, Supapohn Yamuangmorn, Muhammad Yasin Ashraf, Virinder Singh Sohu, Yueqiang Zhang, Fábio Aurélio Dias Martins, Suchada Jumrus, Yusuf Tutus, Mustafa Atilla Yazici, Ismail Cakmak

**Affiliations:** ^1^Agronomy Division, Department of Plant and Soil Sciences, Faculty of Agriculture, Chiang Mai University, Chiang Mai, Thailand; ^2^Pakistan Academy of Sciences, Islamabad, Pakistan; ^3^Department of Plant Breeding & Genetics, Punjab Agricultural University, Ludhiana, India; ^4^Center for Resources, Environment and Food Security, China Agricultural University, Beijing, China; ^5^Department of Soil Science, Federal University of Lavras, Lavras, Brazil; ^6^College of Resources and Environment, Nanjing Agricultural University, Nanjing, China; ^7^Punjab Agricultural University Regional Research Station, Gurdaspur, India; ^8^Soil and Environmental Sciences Division, Nuclear Institute for Agriculture and Biology, Faisalabad, Pakistan; ^9^College of Resources and Environment, Southwest University, Chongqing, China; ^10^Minas Gerais State Agricultural Research Agency, EPAMIG Sul, Campus Universitário UFLA, Lavras, Brazil; ^11^Faculty of Engineering and Natural Sciences, Sabancı University, Istanbul, Turkey

**Keywords:** rice, zinc, iodine, iron, selenium

## Abstract

Widespread malnutrition of zinc (Zn), iodine (I), iron (Fe) and selenium (Se), known as hidden hunger, represents a predominant cause of several health complications in human populations where rice (*Oryza sativa* L.) is the major staple food. Therefore, increasing concentrations of these micronutrients in rice grain represents a sustainable solution to hidden hunger. This study aimed at enhancing concentration of Zn, I, Fe and Se in rice grains by agronomic biofortification. We evaluated effects of foliar application of Zn, I, Fe and Se on grain yield and grain concentration of these micronutrients in rice grown at 21 field sites during 2015 to 2017 in Brazil, China, India, Pakistan and Thailand. Experimental treatments were: (i) local control (LC); (ii) foliar Zn; (iii) foliar I; and (iv) foliar micronutrient cocktail (i.e., Zn + I + Fe + Se). Foliar-applied Zn, I, Fe or Se did not affect rice grain yield. However, brown rice Zn increased with foliar Zn and micronutrient cocktail treatments at all except three field sites. On average, brown rice Zn increased from 21.4 mg kg^–1^ to 28.1 mg kg^–1^ with the application of Zn alone and to 26.8 mg kg^–1^ with the micronutrient cocktail solution. Brown rice I showed particular enhancements and increased from 11 μg kg^–1^ to 204 μg kg^–1^ with the application of I alone and to 181 μg kg^–1^ with the cocktail. Grain Se also responded very positively to foliar spray of micronutrients and increased from 95 to 380 μg kg^–1^. By contrast, grain Fe was increased by the same cocktail spray at only two sites. There was no relationship between soil extractable concentrations of these micronutrients with their grain concentrations. The results demonstrate that irrespective of the rice cultivars used and the diverse soil conditions existing in five major rice-producing countries, the foliar application of the micronutrient cocktail solution was highly effective in increasing grain Zn, I and Se. Adoption of this agronomic practice in the target countries would contribute significantly to the daily micronutrient intake and alleviation of micronutrient malnutrition in human populations.

## Introduction

Globally, an estimated 2 billion people suffer from micronutrient malnutrition (also known as hidden hunger), especially in the developing world. Particular micronutrient deficiencies include Fe, Zn, Se and I which result in severe health complications (Bailey et al., 2015; Harding et al., 2018) as well as significant economic losses (Gödecke et al., 2018). Due to their high requirements, infants, preschool children, and women of reproductive age are more vulnerable to micronutrient malnutrition.

Iron deficiency has been suggested to be the most common micronutrient disorder in human populations, and is often associated with anemia (Bailey et al., 2015). The most prevalent form of anemia is the Fe deficiency anemia which affects over 1 billion people worldwide (Camaschella, 2019). Zinc malnutrition has been found to be responsible for the death of 116,000 children under five years of age, mainly due to enhanced infectious diseases such as diarrhea and pneumonia (Black et al., 2013). Zinc has strong antiviral and antibacterial effects in the human body and is involved in improving immune function and immune response against various viruses, including corona virus (Read et al., 2019; Skalny et al., 2020). Similarly, Se is also required for optimizing the immune system against viral attacks and is required in prevention of various types of cancer in human body and can lower risk of some cancers (Steinbrenner et al., 2015). A large proportion of the world population is known to be at risk of Se malnutrition due to inadequate dietary Se intake (Jones et al., 2017).

Despite the use of iodized salt since decades, human I deficiency has been reported to be still prevalent in a number of countries. An estimated 2 billion people are at risk of I deficiency (Zimmermann and Andersson, 2012; Pearce et al., 2013). Even, some reports indicate that I deficiency is a re-emerging public health problem (Lazarus, 2015; Panth et al., 2019). The well-documented role of I in the human body is related to biosynthesis of thyroid hormones which are important in brain function and mental development (Pearce et al., 2016).

Low availability of soil micronutrients to crop plants and consequent inadequate dietary intake of micronutrients have been discussed as the root cause for the high incidence of micronutrient deficiencies in humans (Welch et al., 2013). According to Welch et al. (2013) crop production systems have never been designed with the aim to improve human nutrition and health which is a further particular reason which partly explains the widespread occurrence of hidden hunger in the world. Therefore, crop production systems should include nutrition-sensitive approaches and be aligned with the production of micronutrient-enriched (biofortified) staple foods for the target populations (Bouis and Saltzman, 2017; Cakmak and Kutman, 2018; Jha and Warkentin, 2020). Most of the staple foods are inherently very low in micronutrients and cannot meet the recommended daily intake of micronutrients (Cakmak, 2008; Andersson et al., 2017; Bouis and Saltzman, 2017), especially rice which is inherently low in micronutrients and is then processed extensively before consumption such as polishing, steaming and parboiling (Balbinoti et al., 2018; Ito and Lacerda, 2019). Today, increasing nutritional value and composition of rice, especially regarding micronutrients is, therefore, a growing challenge.

Among the approaches for enrichment (i.e., biofortification) of micronutrient in staple foods, genetic (i.e., plant breeding) and agronomic (i.e., use of fertilizers) approaches are used widely. After long-term successful efforts, the International HarvestPlus program has developed and released new Zn- or Fe-biofortified wheat and rice varieties containing additional Zn or Fe, at about 10 mg per kg grain (Andersson et al., 2017; HarvestPlus, 2020). Also, multi-country field trials established in the framework of the HarvestZinc project have demonstrated that agronomic biofortification, a fertilizer-based approach, is highly effective in improving grain concentrations of the targeted micronutrients in food crops as shown in wheat, rice, maize and bean for Zn, Se and I (Phattarakul et al., 2012; Zou et al., 2012; Mao et al., 2014; Ram et al., 2016; Cakmak et al., 2017). The impact of the fertilizer-use strategy on grain micronutrient concentration was much more pronounced for foliar in case of the use of foliar sprays, compared with soil applications, for example, Zn in rice plants grown under field conditions (Wissuwa et al., 2007; Phattarakul et al., 2012; Saha et al., 2017). Higher agronomic effectiveness of foliar application over soil application in boosting grain Zn has been also shown for several other crop species (Wang et al., 2012; Zou et al., 2012; Mao et al., 2014; Cakmak and Kutman, 2018).

The contribution of root absorption and root-to-grain transportation of Zn and Fe during the grain filling stage is minimal under field conditions compared to the greenhouse conditions with always high amount of available micronutrients and optimal water (Waters et al., 2009; Kutman et al., 2012; Sperotto et al., 2013; Cakmak and Kutman, 2018). This indicates that mobilization and retranslocation of the already deposited micronutrient reserves within the vegetative tissues to the grains are the key constraints affecting grain accumulation of micronutrients under field conditions. Therefore, maintaining a high pool of micronutrients in the vegetative tissue just before and/or during the grain filling stage, which can be attained with foliar application of micronutrients during grain filling stage, is of great importance and relevance for increasing grain micronutrients (Cakmak and Kutman, 2018). As shown for rice, plant genotypes differ in their genetic capacity to mobilize and translocate Zn from vegetative tissues to grains which needs to be considered in micronutrient biofortification studies (Impa et al., 2013; Johnson-Beebout et al., 2016).

In the present study, we aimed to investigate the foliar applications of Zn, I, Fe and Se alone or together in a cocktail solution on the grain concentrations of these micronutrients in rice grown under different soil and crop management conditions of five major rice growing countries over two years by using seven different rice cultivars. Indeed, there are many publications about the effect of soil and/or foliar applications of micronutrients, usually alone or 2 combined elements, on grain concentration of micronutrients in food crops (Cakmak, 2008; Duffner et al., 2014; Dimkpa and Bindraban, 2016; de Valença et al., 2017; Lyons, 2018). To the best of our knowledge, this is the first study investigating the effect of a cocktail micronutrient solution on grain concentrations of Zn, I, Fe and Se under diverse soil and environmental conditions in five rice growing countries.

## Materials and Methods

### Establishment of Field Experiment

A field experiment on rice was conducted over two cropping seasons in 2015 to 2017 at a total of 21 field sites of five countries, i.e., Brazil, China, India, Pakistan and Thailand. Except for Brazil, where rice was cultivated under upland conditions, in all other locations rice was grown under flooded conditions with 10 to 20 cm standing water above the soil surface for most of the growing season. The rice cultivars grown at various field sites were commonly cultivated in the respective countries (Table 1). Initial soil characteristics of the field locations, including DTPA-extractable soil Zn and Fe measured by Lindsay and Norvell (1978), I determined by Cakmak et al. (2017) and Se measured by Dhillon et al. (2005) are presented in Table 2. The basal fertilizers applied according to local practices of the countries are given in Table 3 and were accomplished before transplanting the crop (except for Brazil, were rice seeds were used instead of seedlings). The given rates of N fertilizer were split-applied and based on respective country’s recommendations as detailed in Table 3.

**TABLE 1:** Location and other variables of the foliar fertilizer experiment in rice grown in wetland conditions conducted at 21 field sites of five countries during 2015–2017.

Country	Year	Field location	Rice cultivar
Brazil	2016	Lambari, and Patos de Minas	BRSMG Caravera
	2017	Lambari, and Patos de Minas	BRSMG Caravera
China	2015	Jiangsu	Zhengdao11
	2016	Chongqing	Xiyou19
India	2015	Ludhiana, and Gurdaspur	PR 124
	2016	Ludhiana, and Gurdaspur	PR 121
Pakistan	2016	Gujranwala-I, Sheikhupura-I, and Sialkot	Super Basmati
	2017	Gujranwala-II, Sheikhupura-II, and Sahiwal	Super Basmati
Thailand	2015	CMU, and Maehia	SPT1
	2016	CMU, and Maehia	SPT1

**TABLE 2:** Initial soil characteristics of 21 experimental field sites in 14 locations of five countries.

Country	Location	pH	DTPA Zn	DTPA Fe	Extractable P	Extractable K	TMAH extractable I	Extractable Se
			(mg kg^–1^)		(mg kg^–1^)	(mg kg^–1^)	(mg kg^–1^)	(μg kg^–1^)
Brazil	Lambari	5.5	1.1	176	39	54	2.9	12.7
	Patos de Minas	5.8	0.7	43	58	144	11.6	9.1
China	Jiangsu	7.5	1.2	115	8.4	80	0.7	10.8
	Chongqing	7.5	0.6		10.1	138	0.9	21.7
India	Ludhiana	7.7	3.7	84	20	178	0.9	15.1
	Gurdaspur	7.8	1.2	20	22	188	2.2	10.1
Pakistan	Gujranwala-I	8.2	1.5	33	4	41	0.2	26.4
	Sheikhupura-I	8.2	2.0	49	18	77	0.6	12.1
	Sialkot	8.1	2.1	113	26	77	0.9	14.0
	Gujranwala-II	8.1	0.6	14	8	51	0.5	8.6
	Sheikhupura-II	8.3	1.7	22	26	77	1.0	20.2
	Sahiwal	8.0	0.9	17	30	80	0.2	8.6
Thailand	CMU	5.5	1.3	51	66	246	0.5	12.0
	Maehia	5.6	0.7	55	113	32	0.7	11.5

*Extractable I was determined by Tetra methyl ammonium hydroxide (TMAH) method by Zia et al. (2015) and Cakmak et al. (2017), extractable Se was determined by Dhillon et al. (2005), extractable Zn and Fe were determined by DTPA, extractable P by Bray-II and extractable K by NaHCO_3_.*

**TABLE 3:** Crop management practices at 14 locations of five countries where the fertilizer experiments were conducted.

Country	Location	Basal fertilizers (kg ha^–1^)			Timing of N application
		N	P	K	
Brazil	Lambari	70/64	40/75	40/45*	1/3 at planting and 2/3 at early jointing
	Patos de Minas				
China	Jiangsu	240	90	90	1/3 at planting, 1/3 at early tillering, 1/6 at booting and 1/6 at flowering
	Chongqing	150	80	75	3/5 at planting and 2/5 at panicle initiation
India	Ludhiana	150	40	30	1/3 at transplanting, 1/3 after 21 days of transplanting and 1/3 after 42 days of transplanting
	Gurdaspur				
Pakistan	Gujranwala-I	130	90	0	1/2 at transplanting, 1/4 after 25 days of transplanting and 1/4 after 50 days of transplanting
	Sheikhupura-I				
	Sialkot				
	Gujranwala-II				
	Sheikhupura-II				
	Sahiwal				
Thailand	CMU	100	31	0	1/2 each at maximum tillering and booting stages
	Maehia				

* N, P, and K basal rates in Brazil are shown for the years 2016/2017, respectively.

### Experimental Design, Foliar Applications and Crop Harvesting

The experiment comprised four treatments: i) local control (i.e., basal N, P, K fertilizers only, no micronutrient applied); ii) local control + foliar application of Zn (0.5% ZnSO_4_⋅7H_2_O in spray solution); iii) local control + foliar application of I (0.05% KIO_3_ in spray solution); and iv) local control + simultaneous foliar spray of micronutrients (Zn, I, Fe and Se) in a cocktail solution containing 0.5% ZnSO_4_⋅7H_2_O + 0.05% KIO_3_ + 0.02% Fe-EDTA + 0.001% Na_2_SeO_4_. The field experiment was laid out in a randomized complete block design with four replications. The foliar solution of each treatment, varying between 500 to 800 L ha^–1^, was applied twice, i.e., at the panicle initiation (about one week prior to heading) and the early grain milk stages. The foliar treatments were applied at late afternoon time until the solution started to run-off from the leaves as described by Cakmak et al. (2010). At grain maturity, rice grain yield was recorded from 6 to 10 m^2^ central area of each plot.

### Chemical Analyses

Subsamples of brown rice (whole caryopsis, without husk) from each plot were secured for analysis of Zn, I, Fe and Se. Brown rice samples were rinsed and washed with distilled de-ionized (DDI) water and dried first with tissue papers and then at about 45 °C in a forced-draft oven to constant weight. Dried brown rice grains were digested in HNO_3_-H_2_O_2_ mixture in a microwave accelerated reaction system (CEM Corp., United States) for the determination of Zn, Fe and Se. For I analysis, brown rice samples were extracted in TMAH at 90 °C using a closed-vessel microwave reaction system (CEM Corp., United States) as described earlier by Zia et al. (2015) and Cakmak et al. (2017). Zinc and Fe concentrations in the digested solutions were measured by inductively coupled plasma optical emission spectroscopy (ICP-OES), and I and Se concentrations by inductively coupled plasma mass spectrometry (ICP-MS). Measurement of mineral nutrients was checked by using certified standard reference materials obtained from the National Institute of Standards and Technology (Gaithersburg, MD, United States).

### Statistical Analysis

The significance of the effects of different foliar treatments on the dependent variables was determined using one-factor ANOVA at 0.05 probability level of least significant difference (LSD) test using SAS software (SAS 8.0, United States). For overall effectiveness, the data sets across locations and years were compared by the paired t test using SPSS 13.0 for Windows. The linear models were used to evaluate correlations among various parameters using MS Excel.

## Results

### Grain Yield

With the same experimental treatments, rice grain (paddy) yields varied drastically across 21 field sites across 14 locations in five countries. For example, in case of the local control treatment (i.e., no micronutrient application), rice grain yield varied from 1.94 Mg ha^–1^ at Lambari location of Brazil in 2017 to 11.46 Mg ha^–1^ at Jiangsu location of China in 2015 (Table 4). Foliar application of Zn, I or micronutrient cocktail did not affect rice grain yield at any field site in any country. Across all experimental treatments and field sites in five countries, average grain yield was 6.10 Mg ha^–1^ with the local control treatment, 6.26 Mg ha^–1^ with the foliar Zn treatment, 5.95 Mg ha^–1^ with the foliar I treatment, and 6.06 Mg ha^–1^ with the micronutrient cocktail treatment (Table 4).

**TABLE 4:** Grain yield of rice grown with different foliar fertilizer treatments at 21 field sites in 14 locations of five countries.

Country	Location	Year	Grain yield (Mg ha^–1^)				*F*-test at *p* < 0.05
			Local control	Foliar Zn	Foliar I	Foliar cocktail	
Brazil	Lambari	2016	2.23	2.35	2.15	2.27	NS
		2017	1.94	1.88	2.10	2.33	NS
	Patos de Minas	2016	4.55	5.30	5.02	5.52	NS
		2017	5.98	5.53	4.86	5.09	NS
China	Jiangsu	2015	11.46	11.11	11.62	11.24	NS
		2016	9.34	9.61	9.56	9.28	NS
	Chongqing	2016	10.73	10.08	10.90	10.39	NS
India	Ludhiana	2016	8.08	8.36	7.84	8.31	NS
		2017	8.33	8.26	7.68	7.61	NS
	Gurdaspur	2016	8.03	8.05	7.92	7.98	NS
		2017	8.41	8.38	8.28	8.15	NS
Pakistan	Gujranwala-I	2016	5.42	Nd	5.40	5.56	NS
	Sheikhupura-I	2016	4.61	Nd	4.48	4.67	NS
	Sialkot	2016	5.51	Nd	5.41	5.51	NS
	Gujranwala-II	2017	4.56	4.81	4.47	4.46	NS
	Sheikhupura-II	2017	4.59	4.70	4.73	4.94	NS
	Sahiwal	2017	4.25	4.28	4.42	4.46	NS
Thailand	CMU	2015	6.51	6.55	6.08	6.07	NS
		2016	4.57	4.13	3.87	4.01	NS
	Maehia	2015	5.01	4.87	4.66	5.20	NS
		2016	4.06	4.54	3.64	4.41	NS
*Mean*			*6.10*	*6.26*	*5.95*	*6.06*	

*Nd, no data (due to an experimental error in the field, grain yield could not be collected). NS, non-significantly different at *P* < 0.05.*

### Grain Zn, I, Fe and Se Concentration

With the local control treatment, brown rice Zn concentration ranged from 15.0 mg kg^–1^ at the Ludhiana field site of India in 2017 to 26.9 mg kg^–1^ at CMU field site of Thailand in 2015 (Table 5). Application of foliar Zn and micronutrient cocktail significantly increased Zn concentration in brown rice at all field sites in all countries (*P <* 0.05), except for at Ludhiana field site of India in both years and at Gurdaspur field site of India in 2016 (Table 5). Average increase in brown rice Zn concentration, across all field sites in five countries, was 31.3% with foliar Zn application and 25.2% with foliar micronutrient cocktail application.

**TABLE 5:** Zinc concentration in brown rice grains grown with different foliar fertilizer treatments in 14 locations of five countries.

Country	Location	Year	Grain Zn concentration (mg kg^–1^)				F-test at *p* < 0.05
			Local control	Foliar Zn	Foliar I	Foliar cocktail	
Brazil	Lambari	2016	22.4 a	28.8 b	23.6 a	28.2 b	*
		2017	25.2 a	32.2 b	25.5 a	30.5 b	*
	Patos de Minas	2016	23.8 a	28.5 b	23.9 a	28.1 b	*
		2017	21.6 a	27.4 b	21.7 a	27.7 b	*
China	Jiangsu	2015	19.3 a	27.8 b	20.9 a	27.1 b	*
		2016	26.0 a	40.5 b	28.1 a	39.8 b	*
	Chongqing	2016	17.8 a	21.4 b	17.8 a	21.4 b	*
India	Ludhiana	2016	21.8	25.8	21.8	21.8	NS
		2017	15.0	22.7	17.0	21.6	NS
	Gurdaspur	2016	21.2	25.0	23.0	23.6	NS
		2017	16.8 a	18.4 b	17.3 a	19.2 b	*
Pakistan	Gujranwala-I	2016	20.9 a	Nd	21.9 a	31.5 b	*
	Sheikhupura-I	2016	19.2 a	Nd	18.7 a	25.3 b	*
	Sialkot	2016	18.7 a	Nd	18.0 a	23.0 b	*
	Gujranwala-II	2017	18.5 a	24.3 b	18.3 a	22.0 b	*
	Sheikhupura-II	2017	18.6 a	23.2 b	18.8 a	22.9 b	*
	Sahiwal	2017	19.0 a	27.3 b	19.6 a	27.3 b	*
Thailand	CMU	2015	26.9 a	31.5 b	26.1 a	33.0 b	*
		2016	27.3 a	30.4 b	25.5 a	27.8 b	*
	Maehia	2015	26.5 a	31.6 b	27.4 a	31.7 b	*
		2016	23.3 a	39.6 b	23.3 a	29.6 b	*
*Mean*			*21.4*	*28.1*	*21.8*	*26.8*	
*% increase in grain Zn concentration over local control treatment*				*31.3%*	*–*	*25.2%*	

Nd, no data (due to an experimental error in the field, grain samples could not be collected). NS, non-significant different at *P* < 0.05. Numbers in the same row followed by different letters differ significantly at LSD_0.05_. * = significantly different at *P* < 0.05.

The range in I concentration in brown rice was wider than the range in Zn concentration. In the case of the local control treatment, I concentration varied from 4 μg kg^–1^ at Ludhiana and Gurdaspur field sites of India in 2016 and 2017, respectively to 34 μg kg^–1^ at Patos de Minas field site of Brazil in 2017. Foliar application of I and micronutrient cocktail resulted in several fold increases in brown rice I concentration at all field sites in all countries (*P* < 0.05; Table 6). On average, across all field sites in all countries, rice grain I concentration was 11 μg kg^–1^ with the local control, 204 μg kg^–1^ with the foliar I, and 181 μg kg^–1^ with the foliar micronutrient cocktail applications.

**TABLE 6:** Iodine concentration in brown rice grains grown with different foliar fertilizer treatments at 21 field sites in 14 locations of five countries.

Country	Location	Year	Grain I concentration (μg kg^–1^)			F-test at *p* < 0.05
			Local control	Foliar I	Foliar cocktail	
Brazil	Lambari	2016	6 a	410 c	249 b	*
		2017	15 a	109 b	148 c	*
	Patos de Minas	2016	5 a	632 c	417 b	*
		2017	34 a	137 c	103 b	*
China	Jiangsu	2015	13 a	96 b	209 c	*
		2016	20 a	276 b	355 c	*
	Chongqing	2016	10 a	28 a	187 b	*
India	Ludhiana	2016	4 a	162 c	94 b	*
		2017	5 a	63 b	133 c	*
	Gurdaspur	2016	9 a	206 c	113 b	*
		2017	4 a	16 ab	25 b	*
Pakistan	Gujranwala-I	2016	18 a	393 c	258 b	*
	Sheikhupura-I	2016	11 a	168 c	121 b	*
	Sialkot	2016	12 a	143 c	125 b	*
	Gujranwala-II	2017	19 a	121 c	101 b	*
	Sheikhupura-II	2017	10 a	177 c	137 b	*
	Sahiwal	2017	13 a	181 c	96 b	*
Thailand	CMU	2015	14 a	254 c	190 b	*
		2016	11 a	194 b	229 c	*
	Maehia	2015	5 a	216 b	202 b	*
		2016	5 a	292 b	313 b	*
*Mean*			*11*	*204*	*181*	

Numbers in the same row followed by different letters differ significantly at LSD_0.05_. * = significantly different at *P* < 0.05.

Variation in Fe concentration in brown rice across all field sites with the local control treatment was greater than the variation in grain Zn concentration. With this treatment, brown rice Fe concentration showed a range between 8.0 mg kg^–1^ at Gurdaspur field site of India in 2016 and 27.8 mg kg^–1^ at Jiangsu field site of China in 2015 (Table 7). However, unlike brown rice Zn density, rice grain Fe concentration was not affected by foliar application of micronutrient cocktail, except for two field sites in Brazil, i.e., Lambari in 2016 and Patos de Minas in 2017. Consequently, average brown rice Fe concentration with all experimental treatments remained almost the same, i.e., 11.8–12.8 mg Fe kg^–1^ grains (Table 7).

**TABLE 7:** Iron concentration in brown rice grains grown with different foliar fertilizer treatments at 21 field sites in 14 locations of five countries.

Country	Location	Year	Grain Fe concentration (mg kg^–1^)				F-test at *p* < 0.05
			Local control	Foliar Zn	Foliar I	Foliar cocktail	
Brazil	Lambari	2016	10.8 a	10.6 a	10.9 a	12.4 b	*
		2017	10.6	12.0	12.3	11.2	NS
	Patos de Minas	2016	15.4	15.8	13.9	16.5	NS
		2017	12.4 a	12.2 a	11.0 b	12.4 a	*
China	Jiangsu	2015	27.8	19.3	19.7	22.8	NS
		2016	10.8	10.3	9.9	11.4	NS
	Chongqing	2016	11.5	14.1	12.5	14.7	NS
India	Ludhiana	2016	8.3	8.9	8.1	8.9	NS
		2017	11.3	12.9	14.0	13.2	NS
	Gurdaspur	2016	8.0	8.1	8.0	6.9	NS
		2017	12.9	11.3	10.7	12.8	NS
Pakistan	Gujranwala-I	2016	17.6	Nd	19.0	18.6	NS
	Sheikhupura-I	2016	12.8	Nd	11.1	12.6	NS
	Sialkot	2016	17.7	Nd	10.3	11.7	NS
	Gujranwala-II	2017	14.7	12.9	12.7	14.7	NS
	Sheikhupura-II	2017	10.5	9.9	11.0	11.4	NS
	Sahiwal	2017	13.9	14.3	14.7	15.2	NS
Thailand	CMU	2015	9.8	9.9	9.4	11.5	NS
		2016	10.4	9.9	9.7	10.7	NS
	Maehia	2015	9.1	9.1	9.2	9.5	NS
		2016	9.9	10.4	11.5	10.4	NS
*Mean*			*12.7*	*11.8*	*11.9*	*12.8*	

Nd, no data; NS, non-significant different at *P* < 0.05. Numbers in the same row followed by different letters differ significantly at LSD_0.05_. * = significantly different at *P* < 0.05.

The Se concentration in brown rice across all field locations showed a large variation with the same micronutrient treatment. With the local control treatment, it was 3 μg Se kg^–1^ at Patos de Minas, Brazil in 2016 to as high as 404 μg Se kg^–1^ at Ludhiana, India in 2017 (Table 8). The foliar application of the micronutrient cocktail resulted in substantial increases in Se concentration in brown rice at all field locations in all countries (*P* < 0.05); the overall magnitude of increase in grain Se concentration was 4-fold (Table 8).

**TABLE 8:** Selenium concentration in brown rice grains grown with different foliar fertilizer treatments at 21 field sites in 14 locations of five countries.

Country	Location	Year	Grain Se concentration (μg kg^–1^)		*F*-test at *p* < 0.05
			Local control	Foliar cocktail	
Brazil	Lambari	2016	9 a	262 b	*
		2017	5 a	172 b	*
	Patos de Minas	2016	3 a	313 b	*
		2017	5 a	352 b	*
China	Jiangsu	2015	32 a	231 b	*
		2016	41 a	562 b	*
	Chongqing	2016	18 a	90 b	*
India	Ludhiana	2016	290 a	543 b	*
		2017	404 a	602 b	*
	Gurdaspur	2016	111 a	279 b	*
		2017	83 a	191 b	*
Pakistan	Gujranwala-I	2016	46 a	519 b	*
	Sheikhupura-I	2016	82 a	420 b	*
	Sialkot	2016	98 a	376 b	*
	Gujranwala-II	2017	58 a	296 b	*
	Sheikhupura-II	2017	53 a	275 b	*
	Sahiwal	2017	71 a	506 b	*
Thailand	CMU	2015	23 a	449 b	*
		2016	134 a	423 b	*
	Maehia	2015	41 a	530 b	*
		2016	36 a	584 b	*
*Mean*			*95 a*	*380 b*	
*fold increase in brown rice grain Se over local control treatment*				*4*	

Numbers in the same row followed by different letters differ significantly at LSD_0.05_. * = Significantly different at *P* < 0.05.

### Relationships Between Rice Grain and Soil Concentrations of Zn, I, Fe and Se

**FIGURE 1:**
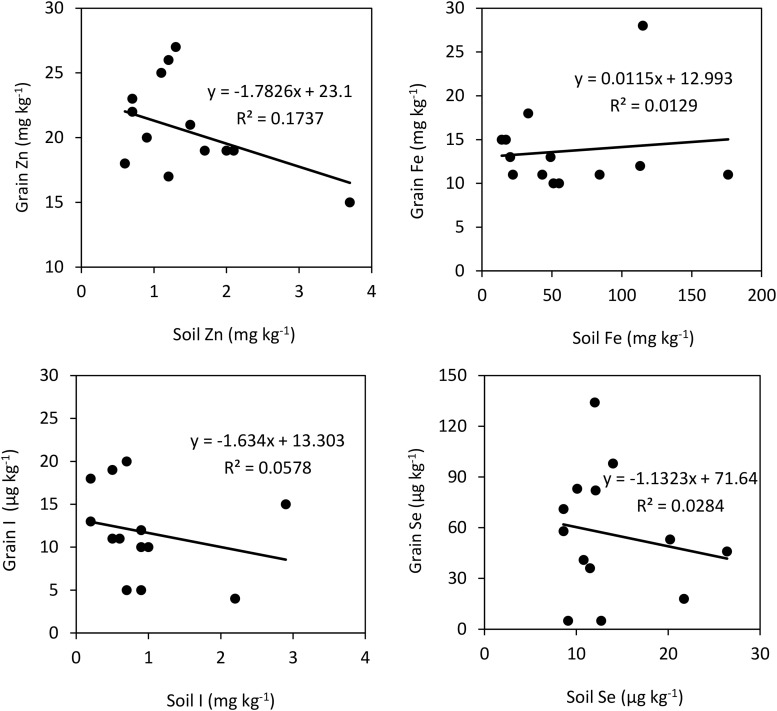
Correlations between soil and grain concentrations of Zn, I, Fe, and Se in plants grown without foliar treatments. Soil Zn and Fe represent their DTPA-extractable concentrations, while soil Se and I are 0.1 M KH_2_PO_4_ extractable and TMAH-extractable concentrations, respectively.

The relationships between soil extractable micronutrient concentrations and their grain concentrations are presented in Figure 1. There was even no correlation between soil extractable concentrations of I, Fe and Se and the concentrations of these micronutrients in rice grain. In fact, there even a negative relation between soil extractable Zn and grain Zn concentration in rice (R^2^ = 0.17, *P* < 0.05).

## Discussion

Results of the present study conducted across 21 field sites over two crop seasons in five major rice-growing countries showed that the foliar application of a micronutrient cocktail solution was highly effective in increasing brown rice grain concentrations of Zn, I and Se. The increases in grain concentration of micronutrients happened without any clear change in grain yield by application of the cocktail solution of micronutrients (Table 4). The soils of all the field sites were not deficient in Zn and Fe for rice, and contained generally high extractable concentrations of Zn and especially Fe (Sims and Johnson, 1991). The DTPA-extractable soil Zn and Fe concentrations ranged between 0.6 to 3.7 mg kg^–1^ and 14 to 176 mg kg^–1^, respectively (Table 2). Although several soil chemical and biological factors are known to affect the interpretation of the effect of soil DTPA-extractable Zn on plant growth, it can be, however, generalized that the rice plants may respond to Zn fertilization positively if the DTPA-extractable Zn is below 0.5 mg kg^–1^ (Sims and Johnson, 1991; Fageria, 2013; Duffner et al., 2014). Also, foliar application of Zn is less effective in increasing grain yield of crops compared with its soil application (Zou et al., 2012). Lack of grain yield increase in response to foliar application of Zn and Fe across these field sites could be attributed to adequate amount of DTPA-extractable Zn and Fe for rice (Table 2).

To our knowledge, this study is the first one showing a very positive effect of combined foliar sprays of Zn, I and Se on grain concentrations of these micronutrients in many rice cultivars grown in several countries. A similar positive effect of the foliar applied micronutrient cocktail solution on grain concentrations of the micronutrients has been also shown in wheat grown in 6 different countries (Zou et al., 2019). In contrast to Zn, I and Se, the concentration of Fe in grain was not affected with the spray of the micronutrient cocktail (Table 7). There was only a minimal increasing trend in grain Fe (by about 8%) by spray of micronutrient cocktail solution. Also, foliar applications of Zn to rice foliage in both single and cocktail solutions were also effective in increasing Zn concentration of brown rice grains at most of the field sites (*P* < 0.05; Table 5). The positive effect of foliar application of Zn alone on grain Zn has been shown already in rice (Phattarakul et al., 2012; Mabesa et al., 2013; Goloran et al., 2019). In the present study, the positive impact of foliar Zn application on grain Zn was consistent during both years at all field sites in five different countries, with the exception of a few field sites in India (Table 5). There was, however, a distinct genotypic variation in response to foliar Zn fertilization. For example, the cultivar Zhengdao11 from the Jiangsu location in China consistently showed the highest response to foliar Zn fertilization over 2 years and exhibited an increase over 50%, while the cultivar Xiyou19 from Chongqing in China showed an increase of around 17% only (Table 5). Both rice cultivars used in India (i.e., PR 124 and PR 121) were less responsive to foliar Zn application in terms of increasing grain Zn. It is well-documented that rice cultivars show a large variation in their ability to translocate and deposit Zn in the grains (Impa et al., 2013; Johnson-Beebout et al., 2016; Sanjeeva Rao et al., 2020).

The weak of relationship between grain Zn and DTPA-extractable soil Zn (Figure 1) indicates that retranslocation of Zn from the vegetative parts to grains, rather than the root Zn uptake, might be a key process affecting grain Zn accumulation in different rice genotypes grown in five countries. In agreement with this suggestion, Mabesa et al. (2013) and Impa et al. (2013) showed that grain Zn accumulation in rice genotypes is under a significant influence of Zn remobilization and transport from vegetative parts to grains. However, the relationship between the concentrations of two mineral nutrients existing in the soil and grain depends not only on their chemical availability, but also on the available amount of other mineral nutrients in soil. It is well documented that there is antagonistic interaction among the mineral nutrients during their root uptake and root-to-shoot transport which greatly affects the leaf and grain composition of mineral nutrients (Marschner, 2012; Rietra et al., 2017). Therefore, the weak correlation between the micronutrients in soil and grain (Figure 1) may also depend on the antagonism between nutrients during root uptake. In case of some rice genotypes grown under greenhouse conditions, grain Zn has been found to be in good correlation with the DTPA-extractable soil Zn (Fageria et al., 2011; Johnson-Beebout et al., 2016). Based on these results, it has been suggested that keeping adequate plant available Zn in the soil medium until grain maturation would contribute greatly to grain Zn accumulation through continued and direct root uptake of Zn with little reliance on retranslocation of Zn from vegetative tissues (Johnson-Beebout et al., 2016; Cakmak and Kutman, 2018). Generally, grain Zn correlates very positively with the amount of Zn in the growth medium if Zn is continuously supplied or remains available to the plant roots until grain maturation, which is very common under controlled greenhouse or growth chamber studies. By contrast, when soil Zn availability to plant roots is limited especially during reproductive growth stage, which is very common under field conditions, grain Zn accumulation largely depends on remobilization of Zn from vegetative tissues (Stomph et al., 2009; Waters et al., 2009; Cakmak and Kutman, 2018). Therefore, it can be suggested that the rice genotypes with higher mobilization and translocation capacity for Zn (and also other micronutrients) from the vegetative tissues to grain are potentially the most promising genotypes for higher grain Zn accumulation.

It was important to note that across all field sites, the average Zn concentration in rice grains with foliar application of Zn alone (i.e., 28.1 mg kg^–1^; Table 5) was very similar to the increase achieved by the cocktail spray of micronutrients (i.e., 26. 8 mg kg^–1^). This result clearly indicates that when applied together with three other micronutrients, the leaf absorption and transportation of Zn in the grain are not seriously affected by the other micronutrients present in the same spray solution. Foliar application of KIO_3_ alone had no effect on rice grain Zn and even tended to increase grain Zn (Table 5). A very similar finding has been also reported in field studies conducted on wheat grown in six countries (Zou et al., 2019). The lack of antagonism between Zn and Se (Mangueze et al., 2018) or Fe and Zn (Wei et al., 2012) has been also shown in rice when these micronutrients are sprayed together in the same spray solution.

Iodine is not an essential micronutrient for higher plants (Marschner, 2012) but, food crops absorb, transport and accumulate I in edible parts (Fuge and Johnson, 2015; Cakmak et al., 2017; Gonzali et al., 2017). There are a few studies indicating that I has some beneficial effects on plant growth, however, I might be also phytotoxic when applied at higher concentrations (Medrano-Macías et al., 2016; Gonzali et al., 2017). In the present study, substantial increases were found in grain I with the foliar application of KIO_3_ (Table 6). This significant effect of KIO_3_ on grain I was consistent over two years across the 21 field sites (*P* < 0.05) and was observed with the application of I alone or in combination with other micronutrients in the cocktail. There were no clear antagonistic effects of other micronutrients on grain I accumulation when sprayed together (Table 6). Our earlier studies showed that foliar application of KIO_3_ to rice grown under field conditions in Brazil and Thailand was effective in increasing grain I concentration, and similar positive effects were also found with the foliar application of KI (Cakmak et al., 2017). These results suggested that I is transported to grains via the phloem channel. There are, however, different opinions and discussions regarding phloem mobility of I. Very recently, Golob et al. (2020) showed that foliar-applied I either in the form of KI or KIO_3_ significantly enhanced I concentration in the tubers of kohlrabi plants, indicating phloem transport of I in the plants. Similar findings related to phloem transportation of I have been also reported by Smoleń et al. (2016) and Landini et al. (2011). By contrast, there are studies showing poor phloem transport of I in the plants such as in young spinach plants (Humphrey et al., 2019) and apple trees (Budke et al., 2020). In case of the studies with apple trees, the increases in I concentration of the fruits, which were kept in plastic bags during the foliar spray, were around 4- to 5-fold compared to the trees without foliar I spray (i.e., from 0.4 μg up to 2.0 μg per 100 g FW) (Budke et al., 2020). These increases were, indeed, very significant; but the dietary relevance seems to be minimal. As indicated earlier, for cereals, at least a part of the enhanced grain I through foliar spray could be also related to a direct fortification (i.e., contamination) of florets and seeds with I via spray solution (Cakmak et al., 2017). The differences among the plant species in their capacity for transporting I from vegetative tissues to the sink organs (i.e., grains, fruits, tubers) are probably related to the plant species-related factors, concentration of I in spray solution and coverage uniformity of the I spray solution on the leaves. The age of the leaves (i.e., sink or source status of the leaves) of the experimental plants used in the short-term studies might be also another factor. Considering published data and reported large gradient in I concentrations between the sink and source organs (Tsukada et al., 2008; Cakmak et al., 2017; Budke et al., 2020), it can be suggested that I is not highly phloem mobile in the plants, like nitrogen, phosphorus or potassium (Marschner, 2012); but it can be considered as moderately phloem mobile as also suggested by Hurtevent et al. (2013). Further studies are required for better clarification and understanding of the phloem mobility of I in different plant species considering source and sink status of the treated leaves and other factors highlighted above.

The results in Table 6 suggest that grain I concentrations were similar between the foliar sprays of I alone and the cocktail of the micronutrients. As mentioned earlier, there is no antagonistic interactions between Zn and I during their transport and grain accumulation when sprayed together in the same solution. Similarly, also I and Se seem to be not antagonistic (Table 6). Also in previous studies, no clear interaction was found between I and Se during their leaf absorption and transportation to wheat grains (Zou et al., 2019), lettuce roots (Smoleń et al., 2014), kohlrabi tubers (Golob et al., 2020) and apple fruits (Budke et al., 2020).

The foliar application of the micronutrient cocktail solution was also effective in increasing grain Se concentration at all field sites in five countries (*P* < 0.05). On average, the increase in grain Se with foliar cocktail treatment was 4-fold (i.e., from 95 μg kg^–1^ to 380 μg kg^–1^; Table 8). Selenium is known to be highly phloem mobile in plants, and therefore foliar spray of Se at relatively low rate is suggested to achieve adequate levels of Se in food crops for human nutrition (Lyons, 2018). Considering the Se concentration in micronutrient cocktail of the present study (i.e., 0.001% Na_2_SeO_4_) and two foliar sprays (@ 600 L cocktail solution ha^–1^) the amount sprayed was about 5 g Se ha^–1^ which resulted in marked increases in grain Se. As rice is a predominant staple cereal in many countries of the world, especially in developing countries, Se-enriched rice through the agronomic biofortification could be a sustainable Se source for the human populations living in Se-deficient regions.

In contrast to Zn, I and Se, foliar spray of the micronutrient cocktail solution did not affect grain Fe concentration compared with the local control treatment, except for one field site in Brazil, i.e., at Lambari in 2016 (*P* < 0.05; Table 7). Usually, there is little change in grain Fe concentration in response to foliar Fe application which was ascribed to poor mobility of Fe in phloem (Aciksoz et al., 2011; El-Jendoubi et al., 2014). In a field experiment conducted on several Fe-rich rice cultivars in China, Yuan et al. (2013) observed an average increase of about 15% in Fe concentration of brown rice with foliar-applied Fe-amino acid complex (Fe-AA) alone, and 33% increase when 1% (w/v) nicotianamine was added in the Fe-AA solution (*P* < 0.05). By applying the same micronutrient cocktail solution used in the present study, Zou et al. (2019) found that grain Fe concentration increased only by 13% in wheat grains grown in 27 locations of 6 countries over 2 years. The effect of foliar Fe sprays on grain Fe reported in the literature is very variable and, usually, of little biological significance (Rengel et al., 1999; Aciksoz et al., 2011; Niyigaba et al., 2019). Due to very low phloem mobility of Fe, transgenic approaches might be useful for enrichment of rice grains with Fe to adequate levels for human nutrition (Wu et al., 2019; Huang et al., 2020).

White rice (polished rice) represents the common form for human consumption; however, its nutritional value is lower than brown rice due to loss of significant amount of minerals during polishing, especially micronutrients such as Fe (Prom-u-thai et al., 2007; Hansen et al., 2012; Balbinoti et al., 2018). Therefore, a decline in the micronutrient concentrations of rice grain is expected after the polishing process, but the extent of the loss depends on rice cultivars, distribution pattern of the mineral nutrients within grain as well as the degree of milling in polishing process (Prom-u-thai et al., 2007; Hansen et al., 2012; Saenchai et al., 2012).

## Conclusion

Foliar application of the cocktail micronutrient solution resulted in significant enrichments of Zn, I and Se in brown rice grains under a wide range of environmental and soil conditions of five major rice producing countries. Based on the data published by Statista GmbH the studied five countries produce almost 300 million metric tons of milled rice which corresponds to about 60% of world production of milled rice per year (Shahbandeh, 2020). Thus, a successful adoption of this agronomic strategy at least in the five major rice producing countries would contribute significantly to the daily micronutrient intake and alleviation of micronutrient malnutrition. Very recent studies using a simulated human digestive system showed that Zn, I and Se in agronomically biofortified foods are sufficiently bioaccessible for use by the human body (Cakmak et al., 2020). In a further human Zn absorption study conducted at ETH-Zurich, agronomically biofortified wheat with Zn made a significant contribution to the dietary Zn absorption in humans (Signorell et al., 2019). These new results indicate an effective transfer of micronutrients from field to gut, and highlight relevance of the fertilizer use strategy in fighting the global burden of micronutrient malnutrition in human populations.

## Data Availability Statement

The raw data supporting the conclusions of this article will be made available by the authors, without undue reservation.

## Author Contributions

All authors listed have made a substantial, direct and intellectual contribution to the work, and approved it for publication.

## Conflict of Interest

The authors declare that the research was conducted in the absence of any commercial or financial relationships that could be construed as a potential conflict of interest.
